# Physicochemical properties of reduced-salt cured pork loin as affected by different freezing temperature and storage periods

**DOI:** 10.5713/ab.21.0320

**Published:** 2021-08-25

**Authors:** Haeun Kim, Koo Bok Chin

**Affiliations:** 1 Department of Animal Science, Chonnam National University, Gwangju 61186, Korea

**Keywords:** Cured Pork Loin, Functional Properties, Pre-rigor, Post-rigor, Reduced-salt

## Abstract

**Objective:**

The objective of this study was to evaluate functional properties of reduced-salt pork meat products made of pre-rigor pork loin treated by different freezing temperatures (−30°C and −70°C) during storage.

**Methods:**

Pre-rigor cured pork loin with 1.0% added salt was compared to post-rigor muscle added with 1.5% salt for pH, color (L*, a*, b*), cooking loss (CL), expressible moisture, warner-Bratzler shear value, thiobarbituric acid reactive substances (TBARS), and volatile basic nitrogen (VBN).

**Results:**

Pre-rigor cured pork loins had higher pH and temperature than post-rigor ones as raw meat (p<0.05). pH values were higher for pre-rigor pork loins than those of post-rigor pork loins (p<0.05). Color values did not different among treatments (p>0.05). No color differences were observed during storage period after cooking (p>0.05). The CL (%) of pre-rigor cured pork loins was the lowest when frozen at −70°C. The TBARS and VBN increased from 8 weeks of storage (p<0.05), but no further changed thereafter (p>0.05). Pre-rigor cured pork loins added with 1.0% salt showed similar characteristics to post-rigor pork loins added with 1.5% salt.

**Conclusion:**

Cured pork loins could be produced using pre-rigor muscle added with 1/3 of the original salt level (1.5%) and could be stored for up to 4 wks of frozen storage, regardless of a frozen temperature of −30°C or −70°C without detrimental effects.

## INTRODUCTION

Previous studies have extensively reported that there is a correlation between high blood pressure and excessive salt intake [1]. Therefore, consumers are increasingly trying to reduce their salt intake through consuming reduced-salt foods and this trend has also been seen in meat products. The salt concentration of 2.0% is a common for meat products, however studies have been reported on strategies to reduce salt level to >2% in processed cured meat products [1]. Reduced salt might affect the qualtity of processed meat products. Salt in processed meat products has various functions. It contributes greatly to its palatability for consumers. Salt also affects the processing stability, cooking yield, and water holding capacity (WHC) of meat products [2]. During the storage process, salt is one of key additives in meat products since it can inhibit the growth of microorganisms. Although reduced-salt meat products are important, the addition of certain amount of salt into meat products is also essential. A substitute for salt in the manufacture of reduced-salt meat products has been extensively studied [3,4]. Research on the use of phosphate has been conducted to improve the WHC of meat products [3]. Alternative salts such as CaCl_2_, KCl, and MgCl_2_ for meat products have also been studied [4]. It is known that pre-rigor carcass after slaughter without being refrigerated can maintain a physiologically active state with higher temperature and pH values than post-rigor carcass. Therefore, it has been reported that meat products made of pre-rigor is superior to those made of post-rigor due to better salt-soluble protein extraction, emulsification capacity, and water-holding capacity [5]. Puolanne and Terrell [6] have reported that the production of frankfurter sausages using a pre-rigor pork with reduced salt content show physicochemical aspects of the product similar to sausages made with post-rigor pork with higher level of salt.

Frozen storage is one of common methods used for preserving meat. However, physicochemical characteristics such as color, lipid oxidation, and WHC of meat and meat products can be degraded or changed by freezing and the duration of storage [7]. As mentioned above, many studies have determined pre-rigor characteristics. However, studies on changes in pre-rigor quality during frozen storage are limited. Therefore, the objective of this study was to determine changes in physicochemical properties and textural properties of cured pork loins after injecting 1% salt brine into pre-rigor loins and 1.5% salt brine into post-rigor loins. In addition, quality changes were evaluated at two storage temperatures (−30°C and −70°C) and storage weeks (0, 4, 8, 12 wks) to determine the potential of manufacturing reduced salt cured loin with pre-rigor pork loins.

## MATERIALS AND METHODS

### Raw meat materials

Pre- and post- rigor pork loins were purchased from a local meat market (Gwangju, Korea). The pre-rigor pork loins were used within one h after slaughter. These loins were used after removing external fat and connective tissues. They were divided into about 350 to 400 g with thicknss of about 4 to 5 cm.

### Preparation of cured pork loin

Table 1 shows compositions of brine solution. Approximately 17% brine solution of the loin weight was injected into the pork loin. The pre-rigor loin was injected with 1.0% salt brine solution and the post-rigor loin loin was injected with 1.5% salt brine solution. After immersing in the brine solution used for injection for 30 min, each pork loin after curing was divided into two parts and stored in two freezers (one at −30°C and one at −70°C). The storage weeks were 0, 4, 8, 12 wks. Prior to evaluation, the cured pork loin was thawed at 4°C for 10 h and cooked at a constant temperature of 75°C in a water bath (WiseBath WB Digital Precise Water Bath, Daihan Scientific., Seoul, Korea) for 30 min until the central temperature reached 71°C. After cooking was completed, loins were cooled on ice for 1 hr and then used for analysis.

### pH and color values (CIE L*, a*, b*)

The pH was measured repeatedly five times for each sample using a pH-meter (MP-120, Mettler-Toledo, Schwarzenbach, Switzerland). Results were derived as average values. Color values of raw meats of post- and pre-rigor and cured pork loins before and after cooking were repeatedly measured six times to obtain the average value. The meat color of cured pork loin was measured using a color meter (CR-10, Minolta Co, LTD, Osaka, Japan) with aperture size of 8 mm and a CIE standard illuminant D65 and 10° observer. Lightness (L* value), redness (a* value), and yellowness (b* value) were recorded.

### Cooking loss and expressible moisture

The cooking loss (CL, %) of cured pork loin was determined using the method of Jauregui et al [8]. Cured pork loins were weighed before and after heating to calculate the CL using the following formula:


$$\text{Cooking loss }(\%)=\frac{\left(\text{Weight before cooking}-\text{Weight after cooking}\right)}{\left(\text{Weight before cooking}\right)}\times 100$$

Expressible moisture (EM, %) values of post-rigor and pre-rigor cured pork loins were measured after cooking according to Wheeler et al [9]. After making the sample into about 1.5 g cubes, three sheets of filter paper (Whatman #3) were cut into 1/4. the sample and centrifuged at 1,660×g for 15 min with a Model VS-5500 centrifuge (Vision Scientific Co., Ltd, Bucheon, Korea). Each sample was measured four times and the average value was calculated. The amount of moisture released from the sample was expressed as g/100 (%).

### Warner-Bratzler shear value

Shear forces of post-rigor and pre-rigor cured pork loins after cooking were measured using an Instron Universal Testing Machine (Model 3344, Canton, MA, USA) according to the procedure of Wheeler et al [9]. Each cured pork loin was cored into a diameter of 1.25 cm in the direction of muscle fiber to prepare measurable samples. Shear force was measured 12 times for each sample and the average value was calculated. Shear force of cured pork loin was measured at a shear load of 500 kg m/s^2^ with a shear blade speed of 300 mm/min. It was expressed in unit of force (kgf) required for cutting.

### Volatile basic nitrogen

Volatile basic nitrogen (VBN) values (mg%) of cured pork loins were measured using the method described by Miwa and Iida [10] with a slight modification. Briefly, 90 mL of double distilled (dd) water was added to about 1 g of the sample and homogenized for one minute with a homogenizer (T 25 basic, Ika Labortechnik, Germany). After filtering the homogenized sample with a filter paper (Whatman #2), 1 mL of the filtrate was reacted with 1 mL of a saturated K_2_CO_3_ solution (50 g/100 g) at 37°C for 2 hrs. After incubation, it was titrated with 0.01 *N* HCl. VBN values of cured pork loin samples were expressed in mg%.

### Thiobarbituric acid reactive substances

Thiobarbituric acid reactive substances (TBARS) were evaluated using a published method [11]. Briefly, minced cooked cured pork loin (2 g) was mixed with 3 mL of 2-thiobarbituric acid (1 g/100 mL) and 17 mL of tricholracetic acid (2.5 g/100 mL). The mixture was then inbuated at 90°C for 20 min in a constant temperature bath (WiseBath WB Digital Precise Water Bath, Daihan Scientific, Korea). The finished sample was kept at room temperature for 1 hr. Then 5 mL of the upper layer of the sample was taken and mixed with 5 mL of chloroform. The mixture was centrifuged at 1,500×g for 5 min minutes using a VS-5000N centrifuge (Vision Scientific Co. Ltd., Korea). After mixing 3 mL of the supernatant of the centrifuged sample with 3 mL of petroleum ether, the mixture was then centrifuged again at 1,500×g for 10 min. The absorbance of the supernatant was measured at 532 nm using a spectrophotometer (UV-1601, Shimadzu, Kyoto, Japan). TBARS value was expressed as mg of malondialdehyde (MDA)/kg of sample using a standard-curve.

### Statistical analysis

Three batches of pre- and post-rigor pork loins were manufactured independently. Results are presented as means± standard deviation. Raw cured pork loins were analyzed at 0, 4, 8, and 12 wks, and they were stored frozen at −30°C and −70°C. Before and after cooking, cured pork loin treatment and different storage wks were used as two factors. Mean difference was performed using a two-way analysis of variance. If there has no interation between treatments and storage time, the data were pooled. Statistical analyses were performed using the SPSS software program version 20 (SPSS Inc., Chicago, IL, USA). Significance was considerd at p<0.05.

## RESULTS AND DISCUSSION

### pH values and temperature of raw post- and pre-rigor pork loin samples

pH values and temperature of post- and pre-rigor pork loin raw meat samples are shown in Table 2. The mean pH value of post-rigor pork loin was 5.54±0.17, which was lower than that (5.80±0.49) of pre-rigor pork loin (p<0.05). The temperature was 14.6°C±6.19°C for post-rigor pork loin and 33.0°C±1.79°C for pre-rigor pork loin. Liu et al [12] reported that the pH values of pre-rigor pork meat within 2 hrs post-mortem were the same as the initial pH when the incubation temperature was less than 40°C. Since pre-rigor muscle is still metabolizing, it has a high temperature and pH value, and has the properties of maintaining functionality such as enhancement of WHC, extraction of salt-soluble protein and improvement of processing yield [5]. Since the pre-rigor pork loin in this study used meat within 2 hrs before rigor completion, and the pH values and temperature were higher than those of the post-rigor pork loin (p<0.05), it was judged that the raw meat of pre-rigor pork loin used in this study had appropriate characteristics of pre-rigor muscles.

### Characteristics of post- and pre-rigor cured pork loins according to storage weeks (0, 4, 8, and 12 wks) and freezing temperature (−30°C and −70°C)

#### pH and color values of post- and pre-rigor cured pork loins before cooking

As shown in Table 3, since there was no effect of interaction between the two factors (storage time and treatment) on pH or meat color values (p>0.05), results of pH values and meat color were expressed by treatment in a storage time or storage in a treatment. Although pH values were different among treatments (p<0.05), pH values of each group showed no differences among storage times (p>0.05). Pre-rigor cured pork loins had pH values from 5.82±0.22 to 5.87±0.20 regardless of the freezing temperature. These values were approximately 0.2 to 0.25 higher than those of post-rigor cured pork loins (p<0.05). Therefore, pre-rigor muscles had higher pH values than post-rigor ones even after injecting a brine solution. Judge and Aberle [13] measured the pH as by according to the salt addition of post- and pre-rigor of minced pork. No differences in pH values were observed according to the presence or absence of salt addition in the same muscle type, but different from post- and pre-rigor. Claus and Sørheim [5] reported that in the pH of the pre-rigor beef patty and post-rigor beef patty with the same salt concentration of 1.7%, the pre-rigor patty was still higher than the post-rigor patty after 1 day of post mortem. This result explained that the addition of salt might delay the glycolysis of the pre-rigor. Song et al [4] noted a higher pH value in pre-rigor chicken breast than post-rigor chicken breast after salt addition. The present study also revealed the same results, showing that pH was affected by the state (pre vs post rigor) of the carcass. When the pork loin was frozen and stored after the brine solution was injected, there was no difference in the pH values between post- and pre-rigor pork loin regardless of freezing storage temperature. In addition, no differences in pH values were observed during the storage time of 12 wks (p>0.05).

Results of color values revealed no significant difference in lightness (L*) or yellowness (b*) according to treatment or storage time (p>0.05). L* values were from 50.6±4.94 to 53.0 ±3.11 for all treatments. They were changed from 50.5±3.09 to 52.4± 4.82 during the storage time. Teuteberg et al [14] found no interation between frozen storage temperature and storage duration.

During the storage times, b* values were from 3.99±0.75 to 4.73 ±1.11 (p>0.05). Redness (a*) values were not significantly different by freezing temperature (p>0.05), but was significantly different by storage time (p<0.05). Redness (a*) values at 8 wks of storage were higher than their initial values. However, after 8 wks, they did not change significantly (p> 0.05). Medić et al [15] found that pork loin showed the lowest redness at 3 months when stored under frozen at −18°C for 18 months, and increased thereafter. Alonso et al [7] also showed that the redness value of frozen pork after two years was lower than those of non-frozen pork. The metmyoglobin proportion increased after two years of frozen storage, whereas the proportion of oxymyoglobin decreased, affecting redness. However, in this study, there was no decreases in redness values during the freezing process. Xia et al [16] reported that the thawing method had an effect on meat color. Thus, it should be taken into account that various factors such as freezing and thawing methods and meat preparation may have influenced the redness values.

#### pH and color values of post- and pre-rigor cured pork loins after cooking

Table 4 shows pH and color results of post- and pre-rigor cured pork loin after cooking during storage times. The pH value was different according to treatmeats and storage tiems (p<0.05). For the same freezing temperature, the pre-rigor pork loin had lower pH value than the post-rigor loin (p<0.05). However, there were no differences in pH among freezing temperatures for the same carcass state (p>0.05). Drerup et al [17] have prepared fresh pork sausages with a salt concentration of 2% using post-rigor, pre-rigor, and a mixture of post-rigor and pre-rigor pork loin and reported that pre-rigor fresh sausages show higher pH than other treatments. The results according to the storage time showed a significant increase at the 8th wk of storage (p<0.05), but the 12th wk did not show any difference from initial storage to 4th wk of storage.

L* value was not affected by the treatment or the storage time (p>0.05). L* values were 73.7±2.14 or higher for all treatments. For a* values, post-rigor cured pork loins showed lower a* values than pre-rigor cured pork loin (p<0.05). However, b* values showed no difference between treatment (p>0.05), although they showed differences by the storage time. Although the 8th wk of storage showed a lower b* value than the initial b* value at the beginning of the storage time (p<0.05), no differences were observed thereafter (p>0.05). Thomas et al [18] reported that a* and b* values of sausages made with pre-rigor pork loins were higher than those made of post-rigor pork loins. They suggested that it might be partially because the content of myoglobin in the pre-rigor pork was lower than that of post-rigor pork. Kim et al [19] reported that the content of myoglobin did not affect lightness and yellowness, but there was a significant correlation between redness and myoglobin. In this study, post-rigor cured pork loins had lower a* values than pre-rigor cured pork loins (p< 0.05), which might be due to highr level of myoglobin content in pre-rigor, resulting in higher a* values.

#### Cooking loss and expressible moisture of post- and pre-rigor cured pork loins after cooking

Effects of freezing temperature and storage time on CL and EM of post- and pre- rigor cured pork loins are shown in Table 5. The CL of post-rigor pork cured loin at storage temperature of −30°C was higher than thoat of pre-rigor cured pork loin at storage temperature of −70°C (p<0.05). Choi and Chin [3] reported that the cooking yield was increased as the salt concentration was increased. Although 1.5% of salt was added to the post-rigor cured pork loin and 1.0% of salt was added to the pre-rigor pork cured loin, the pre-rigor cured pork loin had a lower or similar CL compared to the cured post-rigor pork loin. Karakaya et al [20] found that the high pH of the pre-rigor influenced the decrease in CL, and Mortensen et al [21] also reported that high pH values were associated with CL. No differences in freezing storage temperature for both post- and pre-rigor cured pork loin were observed for CL (p>0.05). This result is in agreement with Bertram et al [22], who found that during storage, fresh pork meats were not affected by CL, depending on the different frozen temperatures of −20°C and −80°C. However, Mortensen et al [21] reported that long term storage following a fast freezing lead to damage to the meat because it distressed the water distribution within the meat, whereas slow freezing was more suitable for meat kept in long term storage than fast freezing method. Choi et al [23] noted that slow freezing rate caused more damage to meat because more ice crystals were formed extracellular than quick freezing method. Utrera et al [24] suggested that the degree of protein oxidation had an influence on the WHC of meat protein. Since there was no differences in the results of VBN according to freezing storage temperature (Table 5), thus it is considered that there is no differences in CL according to the freezing temperature. As a results of the storage time, although initial storage (the date of manufacture) showed the highest CL (p<0.05), there were no differences in CL values from the 4th wk to the 12th wk of storage (p>0.05). Medić et al [15] reported that there was no difference in CL during the storage time except for the day of manufacture, similar to results of this study.

On the other hand, all treatments did not show any differences in EM values (p>0.05). This is different from the results of CL, which may be due to the difference in the source of the liquid lost in each process. CL comes from the degeneration of meat protein by heating, resulting in a decrease in the water binding properties and from the fat that melts during heating. However, the loss due to pressing comes from the constituent water [25]. The EM values of pre-rigor pork cured loins treated with 1.0% salt were similar to those of post-rigor cured pork loins treated with 1.5% salt. This confirmed that the WHC of the pre-rigor cured pork loin was higher than that of the post-rigor cured pork loin. Drerup et al [17] have observed that a high pH of pre-rigor pork loin has a positive effect on the extraction of salt soluble proteins, resulting in increased binding capacity. This suggests that the EM of pre-rigor pork loin is reduced by improving the functional properties due to the high pH of the raw pre-rigor. The EM of post-rigor pork loin was not affected by different freezing temperatures of −30°C and −70°C. The EM values were the same for pre-rigor muscles stored at −30°C and −70°C (p>0.05). Kim et al [26] have reported that freezing method and freezing temperature have no effect on the WHC of pork meat (belly vs loin), unlike other meat types (chicken or beef). Sakata et al [27] noted that there was no significant difference in the WHC of pork stored frozen between −20°C and −80°C, similar to results of the present study. However, Zhang et al [28] have reported that fast frozen pork loins showed higher water holding capacities than slow frozen ones due to protein denaturation caused by the formation of ice crystals during freezing. However, pre-salted meat before freezing shows reduced intracellular damages caused by freezing [29]. There was no difference in EM of pork loins stored at different freezing temperatures. It might be because pork loins applied in this study were cured before freezing in this study. In addition, EM was not changed with increasing storage time (p>0.05). Since the EM content (%) was maintained in the same state as that at the initial storage at −30°C for 12 wks, freezing storage would be suitable for post- and pre- rigor pork loins.

### Warner-Bratzler shear values of post- and pre-rigor pork cured loins after cooking

Table 5 shows results of shear values (SV). No differences in SV between different frozen temperatures (−30°C vs −70°C) were observed (p>0.05). Koohmaraie et al [30] reported that pre-rigor lamb muscle stored at −5°C had similar sarcomere shortening as those stored at −30°C, resulting in similar shear force values. Grujić et al [31] reported that beef muscles frozen at −20°C displayed more non-uniform ice crystals in the intercellular region compared to those at frozen at −78°C, and found that freezing temperatures below −50°C could decrease freezing damage to muscle. However, Jiang et al [29] suggested that salted meat was less damaged from freezing. Since pork loin in this study was pre-salted and then stored frozen, ice crystal formation during storage might be a problem due to pre-salting prior to frozen. Thus, it might not affect the SV of pre-cured pork, depending on the frozen temperature. In addition, rigor state (post-rigor vs pre-rigor) and salt level didn’t affect the SV in this study (p>0.05). The result agreed with Sabikun et al [32] that no differences in SV were found in pre- and post chicken muscle until 60 days of frozen storage at −20°C. Kim et al [33] reported that hardness values of pre-rigor chicken breasts were higher than post-rigor chicken breasts with 2% salt because of the high protein solubility and low myofibril fragmentation index in pre-rigor muscle. In this study, pre-rigor cured pork loin with a salt concentration of 1.0% showed similar SV as post-rigor cured pork loin with 1.5% salt.

The SV decreased from the 4th wk of storage and no further decrease thereafter. This was partially due to the formation of ice crystals generated during the freezing storage, resulting in protein denaturation. Such ice crystals could deform muscle fiber structrure, resulting in an increased tenderness as muscle fibers are compressed and deformed by the pressure of ice crystals [25].

### Volatile basic nitrogen and thiobarbituric acid reactive substances of post- and pre-rigor cured pork loins after cooking

Table 5 shows results of VBN and TBARS. The VBN was not affected by treatment (p>0.05). However, there was a difference according to the storage time. It showed a tendency to increase with longer storage time, especially at 8 wks of storage (p<0.05). When VBN is higher than 30 mg%, spoilage might be progressed. If meat has VBN of 5 to 10 mg%, it is considered as fresh meat [34]. From results of this study, at the 12th wk of storage, the VBN was 8.34±1.59 mg%, indicating that spoilage was not started yet. Since the storage temperature (−30°C and −70°C) did not result in any difference in VBN, it is thought that economic loss can be reduced by freezing meat products for storage.

Results of TBARS did not differ by the treatment (p>0.05). However, they showed differences by the storage time (p< 0.05). It was confirmed that the longer the storage time, the higher the TBARS (p>0.05). After storage for 12 wks at 2 different frozen storage temperatures, the TBARS values were less than 0.57±0.17 mg MDA/kg, which was far below threshold value of TBARS. When the TBARS value is more than 0.6 mg MDA/kg, consumers generally might detect an off-flavor [35]. Since the results of this study showed a value of 0.57±0.17 mg MDA/kg until 12 wks of storage, it confirmed that lipid oxidation might be delayed by frozen storage, and the quality of cured pork loin due to lipid oxidation was not changed. In addition, there was no difference in TBARS by storage temperature (p>0.05), indicating that freezing storage at −30°C could be as good as freezing storage at −70°C. There was no differences in TBARS between pre- and post-rigor, which was a similar result to those of Drerup et al [17].

## CONCLUSION

Cured pork loins manufactured with pre-rigor pork loins treated with 1.0% salt had properties similar to cured pork loin manufactured with post-rigor pork loins treated with 1.5% salt. Therefore, it is possible to manufacture reduced-salt cured pork using pre-rigor pork loins. The frozen storage of cured pork loins up to 4 wks did not change the quality loss if the pork loins were cured prior to frozen, regardless of the frozen temperatures (−30°C vs −70°C). Further studies are needed to determine how to enhance the falvor and taste of reduced-salt meat products.

## Figures and Tables

**Table 1:** Formulation of post-rigor and pre-rigor pork loins treated with brine solution

Items	Rigor state^1)^	

	Post rigor	Pre rigor
Ingredients		
Pork loin	83.3	83.3
Ice water (%)	12.7	12.7
Salt (%)	1.30	0.80
Phosphate (%)	0.40	0.40
Cure blend^2)^ (%)	0.25	0.25
Sodium erythorbate (%)	0.05	0.05
Sugar (%)	1.00	1.00
Corn syrup solid (%)	1.00	1.00
Total	100	99.5

1) Rigor state: post rigor, loin ham manufactured with post-rigor pork loin containing 1.5% salt; pre rigor, loin ham manufactured with pre-rigor pork loin containing 1.0% salt.

2) Cure blend, containing 93.75% NaCl and 6.25% NaNO_2_.

**Table 2:** pH and color values of raw pork loins

Items	Post-rigor	Pre-rigor
pH	5.54±0.17^b^	5.80±0.49^a^
Temperature	14.6±6.19^b^	33.0±1.79^a^

a,b Means with different superscripts in a same row are different at p<0.05.

**Table 3:** pH and color values of post- and pre-rigor raw pork loins stored at different freezing temperatures

Item	Parameters			

	pH	L*	a*	b*
Treatment	^**^	NS	NS	NS
Storage time (wks)	NS	NS	^**^	NS
Treatment×storage time (wks)	NS	NS	NS	NS
Treatment^1)^				
post rigor-30	5.63±0.11^b^	51.6±3.26^a^	5.27±1.95^a^	4.28±1.26^a^
post rigor-70	5.62±0.12^b^	53.0±3.11^a^	5.23±1.69^a^	4.45±1.39^a^
pre rigor-30	5.87±0.20^a^	51.0±4.03^a^	4.50±1.61^a^	4.03±1.01^a^
pre rigor-70	5.82±0.22^a^	50.6±4.94^a^	4.66±2.00^a^	4.57±0.90^a^
Storage time (wks)				
0	5.72±0.32^a^	51.7±4.64^a^	2.81±0.59^b^	4.73±1.11^a^
4	5.73±0.16^a^	50.5±3.09^a^	4.69±0.96^ab^	3.99±0.75^a^
8	5.72±0.14^a^	51.6±4.38^a^	5.20±1.72^a^	4.54±1.03^a^
12	5.84±0.18^a^	52.4±4.82^a^	6.82±2.53^a^	4.07±1.50^a^

NS, not significant.

1) Treatments: post rigor-30, Loin ham manufactured with post-rigor loin containing 1.5% salt and stored frozen at −30°C; post rigor-70, Loin ham manufactured with post-rigor pork loin containing 1.5% frozen storage at −70°C; pre rigor-30, Loin ham manufactured with pre-rigor pork loin containing 1.0% salt and stored frozen at −30°C; pre rigor-70, Loin ham manufactured with pre-rigor containing 1.0% salt and frozen storage at −70°C.

a,b Means of treatment or storage time with different superscripts in a same column are statistically different at p<0.05.

** p<0.05.

**Table 4:** pH and color values of cooked pork loins made with post- and pre-rigor pork loins stored at different freezing temperatures

Items	Parameters			

	pH	L*	a*	b*
Treatment	^**^	NS	^**^	NS
Storage time (wks)	^**^	NS	NS	^**^
Treatment×storage time (wks)	NS	NS	NS	NS
Treatment^1)^				
post rigor-30	5.85±0.10^b^	74.4±2.44^a^	7.50±0.09^b^	4.49±1.23^a^
post rigor-70	5.85±0.11^b^	76.2±1.55^a^	7.47±0.21^b^	4.10±1.71^a^
pre rigor-30	6.00±0.08^a^	73.7±2.14^a^	8.45±0.72^a^	4.80±0.79^a^
pre rigor-70	5.98±0.09^a^	74.0±2.00^a^	8.28±1.32^a^	5.03±1.17^a^
Storage time (wks)				
0	5.89±0.14^b^	75.0±2.51^a^	8.53±1.14^a^	5.15±0.27^a^
4	5.86±0.10^b^	74.5±1.63^a^	7.53±1.05^a^	4.43±0.81^ab^
8	5.90±0.08^a^	74.3±2.51^a^	7.67±1.40^a^	3.51±1.78^b^
12	5.92±0.08^ab^	74.5±2.20^a^	7.97±1.03^a^	5.33±0.87^ab^

NS, not significant.

1) Treatments: post rigor-30, Loin ham manufactured with post-rigor loin containing 1.5% salt and stored frozen at −30°C; post rigor-70, Loin ham manufactured with post-rigor pork loin containing 1.5% frozen storage at −70°C; pre rigor-30, Loin ham manufactured with pre-rigor pork loin containing 1.0% salt and stored frozen at −30°C; pre rigor-70, Loin ham manufactured with pre-rigor containing 1.0% salt and frozen storage at −70°C.

a,b Means of treatment or storage time with different superscripts in a same column are statistically different at p<0.05.

** p<0.05.

**Table 5:** Cooking loss (CL, %), expressible moisture (EM, %), shear value (SV, %), thiobarbiturcic acid reactant sustances (TBARS), and volatile basic nitrogen (VBN, mg%) of cooked pork loins made with post- and pre-rigor pork loin during frozen storage

Items	Parameters				

	CL (%)	EM (%)	SV (kgf)	TBARS	VBN
Treatment	^**^	NS	NS	NS	NS
Storage time (wks)	^**^	NS	^**^	^**^	^**^
Treatment×storage time (wks)	NS	NS	NS	NS	NS
Treatment^1)^					
post rigor-30	23.1±3.00^a^	27.3±2.47^a^	2.79±0.63^a^	0.49±0.13^a^	6.44±2.28^a^
post rigor-70	21.2±4.49^ab^	26.4±3.93^a^	2.71±0.73^a^	0.50±0.13^a^	6.01±2.19^a^
pre rigor-30	20.8±2.44^ab^	25.0±2.95^a^	2.73±0.51^a^	0.49±0.12^a^	5.89±2.17^a^
pre rigor-70	19.8±3.53^b^	25.1±2.20^a^	2.64±0.63^a^	0.48±0.12^a^	5.44±1.93^a^
Storage time (wks)					
0	26.0±3.80^a^	25.0±2.80^a^	3.27±0.47^a^	0.40±0.11^c^	4.30±0.62^c^
4	22.2±3.24^b^	26.4±3.58^a^	2.36±0.52^b^	0.45±0.04^bc^	4.01±0.60^c^
8	19.5±4.24^b^	25.5±2.97^a^	2.52±0.50^b^	0.52±0.17^ab^	6.72±1.28^b^
12	18.4±6.84^b^	26.6±2.69^a^	2.72±0.57^ab^	0.57±0.17^a^	8.34±1.59^a^

NS, not significant.

1) Treatments: post rigor-30, Loin ham manufactured with post-rigor loin containing 1.5% salt and stored frozen at −30°C; post rigor-70, Loin ham manufactured with post-rigor pork loin containing 1.5% frozen storage at −70°C; pre rigor-30, Loin ham manufactured with pre-rigor pork loin containing 1.0% salt and stored frozen at −30°C; pre rigor-70, Loin ham manufactured with pre-rigor containing 1.0% salt and frozen storage at −70°C.

a–c Means of treatment or storage time with different superscripts in the same column are significantly different at p<0.05.

** p<0.05.

## References

[1] Bhat ZF, Morton JD, Mason SL, Bekhit AEDA (2020). The application of pulsed electric field as a sodium reducing strategy for meat products. Food Chem.

[2] Desmond E (2006). Reducing salt: A challenge for the meat industry. Meat Sci.

[3] Choi JS, Chin KB (2020). Evaluation of physicochemical and textural properties of chicken breast sausages containing various combinations of salt and sodium tripolyphosphate. J Anim Sci Technol.

[4] Song DH, Ham YK, Ha JH, Kim YR, Chin KB, Kim HW (2020). Impacts of pre-rigor salting with KCl on technological properties of ground chicken breast. Poult Sci J.

[5] Claus JR, Sørheim O (2006). Preserving pre-rigor meat functionality for beef patty production. Meat Sci.

[6] Puolanne EJ, Terrell RN (1983). Effects of Salt Levels in Prerigor Blends and Cooked Sausages on Water Binding, Released Fat and pH. J Food Sci.

[7] Alonso V, Muela E, Tenas J, Calanche JB, Roncalés P, Beltrán JA (2016). Changes in physicochemical properties and fatty acid composition of pork following long-term frozen storage. Eur Food Res Technol.

[8] Jauregui CA, Regenstein JM, Baker RC (1981). A simple centrifugal method for measuring expressible moisture, a water-binding property of muscle foods. J Food Sci.

[9] Wheeler TL, Shackelford SD, Koohmarrie M (2000). Variation in proteolysis, sarcomere length, collagen content, and tenderness among major pork muscles. J Anim Sci.

[10] Miwa K, Iida H (1973). Studies on ethylalcohol determination in “Shiokara” by the microfiltration method. Fish Sci.

[11] Sinnhuber RO, Yu TC (1977). The 2-thiobarbituric acid reaction, an objective measure of the oxidative deterioration occurring in fats and oils. J JOCS.

[12] Liu J, Ruusunen M, Puolanne E, Ertbjerg P (2014). Effect of pre-rigor temperature incubation on sarcoplasmic protein solubility, calpain activity and meat properties in porcine muscle. LWT-Food Sci Technol.

[13] Judge MD, Aberle ED (1980). Effect of prerigor processing on the oxidative rancidity of ground light and dark porcine muscles. J Food Sci.

[14] Teuteberg V, Kluth IK, Ploetz M, Krischek C (2021). Effects of duration and temperature of frozen storage on the quality and food safety characteristics of pork after thawing and after storage under modified atmosphere. Meat Sci.

[15] Medić H, Kušec ID, Pleadin J (2018). The impact of frozen storage duration on physical, chemical and microbiological properties of pork. Meat Sci.

[16] Xia X, Kong B, Liu J, Diao X, Liu Q (2012). Influence of different thawing methods on physicochemical changes and protein oxidation of porcine longissimus muscle. LWT-Food Sci Technol.

[17] Drerup DL, Judge MD, Aberle ED (1981). sensory properties and lipid oxidation in prerigor processed fresh pork sausage. J Food Sci.

[18] Thomas R, Anjaneyulu ASR, Kondaiah N (2008). Effect of hot-boned pork on the quality of hurdle treated pork sausages during ambient temperature (37±1°C) storage. Food Chem.

[19] Kim GD, Jeong JY, Hur SJ, Yang HS, Jeon JT, Joo ST (2010). The relationship between meat color (CIE L* and a*), myoglobin content, and their influence on muscle fiber characteristics and pork quality. Korean J Food Sci An.

[20] Karakaya M, Saricoban C, Yilmaz MT (2005). The effect of various types of poultry pre- and post-rigor meats on emulsification capacity, water-holding capacity and cooking loss. Eur Food Res Technol.

[21] Mortensen M, Andersen HJ, Engelsen SB, Bertram HC (2006). Effect of freezing temperature, thawing and cooking rate on water distribution in two pork qualities. Meat Sci.

[22] Bertram HC, Andersen RH, Andersen HJ (2007). Development in myofibrillar water distribution of two pork qualities during 10-month freezer storage. Meat Sci.

[23] Choi EJ, Park HW, Chung YB, Park SH, Kim JS, Chun HH (2017). Effect of tempering methods on quality changes of pork loin frozen by cryogenic immersion. Meat Sci.

[24] Utrera M, Morcuende D, Estévez M (2014). Temperature of frozen storage affects the nature and consequences of protein oxidation in beef patties. Meat Sci.

[25] Vieira C, Diaz MT, Martínez B, García-Cachán MD (2009). Effect of frozen storage conditions (temperature and length of storage) on microbiological and sensory quality of rustic crossbred beef at different states of ageing. Meat Sci.

[26] Kim GD, Jung EY, Lim HJ, Yang HS, Joo ST, Jeong JY (2013). Influence of meat exudates on the quality characteristics of fresh and freeze-thawed pork. Meat Sci.

[27] Sakata R, Oshida T, Morita H, Nagata Y (1995). Physico-chemical and processing quality of porcine M. longissimus dorsi frozen at different temperatures. Meat Sci.

[28] Zhang Y, Ertbjerg P (2019). On the origin of thaw loss: Relationship between freezing rate and protein denaturation. Food Chem.

[29] Jiang Q, Jia R, Nakazawa N, Hu Y, Osako K, Okazaki E (2019). Changes in protein properties and tissue histology of tuna meat as affected by salting and subsequent freezing. Food Chem.

[30] Koohmaraie M, Doumit ME, Wheeler TL (1996). Meat toughening does not occur when rigor shortening is prevented. J Anim Sci.

[31] Grujić R, Petrović LJ, Pikula B, Amidžić L (1993). Definition of the optimum freezing rate—1. Investigation of structure and ultrastructure of beef M. longissimus dorsi frozen at different freezing rates. Meat Sci.

[32] Sabikun N, Bakhsh A, Ismail I, Hwang YH, Rahman MS, Joo ST (2019). Changes in physicochemical characteristics and oxidative stability of pre- and post-rigor frozen chicken muscles during cold storage. J Food Sci Technol.

[33] Kim HW, Hwang KE, Song DH (2015). Effect of pre-rigor salting levels on physicochemical and textural properties of chicken breast muscles. Korean J Food Sci An.

[34] APFQIA: Animal, Plant and Fisheries Quarantine and Inspection Agency Standard for Processing & Ingredient Specifications of Livestock Product [Internet].

[35] Georgantelis D, Ambrosiadis I, Katikou P, Blekas G, Georgakis SA (2007). Effect of rosemary extract, chitosan and α-tocopherol on microbiological parameters and lipid oxidation of fresh pork sausages stored at 4°C. Meat Sci.

